# Inferring the absence of an incipient population during a rapid response for an invasive species

**DOI:** 10.1371/journal.pone.0204302

**Published:** 2018-09-27

**Authors:** Amy A. Yackel Adams, Björn Lardner, Adam J. Knox, Robert N. Reed

**Affiliations:** 1 U.S. Geological Survey, Fort Collins Science Center, Fort Collins, Colorado, United States of America; 2 Department of Fish, Wildlife, and Conservation Biology, Colorado State University, Fort Collins, Colorado, United States of America; 3 U.S. Geological Survey, Brown Treesnake Project, Dededo, Guam, United States of America; USDA Forest Service, UNITED STATES

## Abstract

Successful eradication of invasives is facilitated by early detection and prompt onset of control. However, realizing or verifying that a colonization has occurred is difficult for cryptic species especially at low population densities. Responding to the capture or unconfirmed sighting of a cryptic invasive species, and the associated effort to determine if it indicates an incipient (small, localized) population or merely a lone colonizer, is costly and cannot continue indefinitely. However, insufficient detection effort risks erroneously concluding the species is not present, allowing the population to increase in size and expand its range. Evidence for an incipient population requires detection of ≥1 individual; its absence, on the other hand, must be inferred probabilistically. We use an actual rapid response incident and species-specific detection estimates tied to a known density to calculate the amount of effort (with non-sequential detections) necessary to assert, with a pre-defined confidence, that invasive brown treesnakes are absent from the search area under a wide range of hypothetical population densities. We illustrate that the amount of effort necessary to declare that a species is absent is substantial and increases with decreased individual detection probability, decreased density, and increased level of desired confidence about its absence. Such survey investment would be justified where the cost savings due to early detection are large. Our Poisson-based model application will allow managers to make informed decisions about how long to continue detection efforts, should no additional detections occur, and suggests that effort to do so is significantly higher than previously thought. While our model application informs how long to search to infer absence of an incipient population of brown treesnakes, the approach is sufficiently general to apply to other invasive species if density-dependent detection estimates are known or reliable surrogate estimates are available.

## Introduction

Invasive species can have dramatic ecosystem impacts and result in invasion debts [1], both of which incur high societal costs [2]. Removal of all individuals of a potentially invasive species may be possible if detection and control are initiated soon after its introduction [3–6]. Early detection and rapid response (EDRR) programs for potentially invasive species commonly consist of systematic attempts to detect incipient populations, followed by eradication or containment efforts if detected [7]. Typical EDRR practice is to survey defined areas for species presence via one or more visits to the location until the target species is detected or until searches cease, once there are repeated failures to detect the target species. Ideally, intensity of the response should be dictated by the degree of certainty of species absence chosen by resource managers, but in practice availability of funding and personnel usually dictate the intensity of response. Managers are often unable to make optimal decisions because they lack the means for determining when their search efforts have achieved their selected level of certainty.

Many rapid response efforts rely on intensive initial searches of short duration after detection of an invasive species. Sometimes these efforts are followed by periodic searches over longer time periods (e.g., [3, 8]) to try and detect the species of concern. For instance, Hodgkins and Foster [4] reported on the accidental introduction of Italian wall lizards, *Podarcis siculus*, in a shipment of stone to the UK from Italy. The initial rapid response captured 4 lizards in a two-day effort. Afterward, the imported stone shipment and surrounding area were placed under monthly surveillance for two years with no additional lizard detections. Decisions on effort duration were not discussed in the paper and presumably were based on expert opinion combined with a good-faith effort. Such incidents emphasize the need to develop analytical and decision making tools for EDRR [7].

Containment, suppression, and local eradication of invasive brown treesnakes (*Boiga irregularis*) on Guam are top management priorities because of the ecological damage that has occurred on the island, and the prospect of similar damage occurring if the snake were to be accidentally transported to other islands. The brown treesnake Rapid Response Team (BTS RRT, coordinated by the U.S. Geological Survey) has been deployed >23 times since 2002 in response to credible snake sightings or captures on snake-free islands in the western Pacific Ocean. These search efforts have never resulted in confirmation of any incipient population, but have always raised the question of when search efforts should cease.

On 03 September 2014 the BTS RRT deployed to the snake-free Pacific island of Rota in response to an immature brown treesnake capture in a mouse-baited early detection snake trap by the seaport [9]. Was the captured snake merely a lone stowaway from Guam, or possibly the tip of a hard-to-detect iceberg? No population was discovered, but the question of when to stop the search arose. While this survey was still ongoing, we developed an objective means for determining the amount of search effort required to meet resource managers’ desired degree of certainty that a snake population was not present in the search area. Here we use the Rota EDRR data to populate a model inferring the absence of any incipient population during a rapid response. Our framework is based on a Poisson distribution with novel inputs of species- and density-specific detection probability estimates for each detection method (visual search and trapping), along with the projected or final level of survey effort.

## Materials and method

### Model parameters and assumptions

The probability of detecting an incipient population is largely dependent on intensity of search effort and the population density at sites being searched. Our model assumes that there is indeed a snake population present in the search area, and quantifies the probability that we would then find at least one snake, given (1) the effort expended, and (2) the snake population density at the sites where we conduct searches. For highly invasive species this probability should be as high as possible, ideally ≥95%.

We model the probability of finding ≥1 snake(s) based on three parameters: the potential snake population density in the surveyed area, level of effort, and detection probability estimates (for individual snakes) associated with the capture methods (visual search and trapping) used. The latter estimates are based on data from a fully censused population of marked and released snakes [10, 11]. In this paper, we apply our model to the visual search effort on Rota with similar calculations for the trapping effort provided online (see S1 Table and S1 Text).

### Application data

The brown treesnake is a notorious invasive species that was accidentally introduced to the U.S. territory of Guam shortly after World War II. From a founder population of ≤10 snakes, and possibly just one gravid female [12], it spread across the island within a few decades [13]. This slender, secretive, nocturnal, and largely arboreal snake is present in all habitats on Guam, from forests to grasslands. It can reach an adult snout-vent length of 2.3 m, but is more commonly <1.3 m. The snake has caused the extinction of most native forest birds, numerous power outages, and severe cases of envenomation among young children [13, 14].

Guam is a transportation hub of the Western Pacific and there is risk of the snake spreading and causing similar damage elsewhere. In the past two decades, numerous brown treesnake captures and credible sightings have occurred on other islands in the Pacific and Indian Oceans, in North America, and as far away as Spain; all are believed to be snakes originating as stowaways in aircraft, ships, and cargo from Guam [15]. To minimize the risk to locales with transportation links to Guam, the United States spends several million dollars annually to inspect outbound cargo from Guam and remove stowaway snakes [15]. Snake interdiction programs also exist in Hawai’i and the Commonwealth of the Northern Mariana Islands, which are on the ‘receiving’ end of the transportation network.

In 2002, the BTS RRT was created to respond to snake sightings/captures on snake-free islands in the region, to help prevent establishment of brown treesnake populations [15]. The BTS RRT uses an incident command structure when responding to credible snake sightings (or captures) and conducts visual searches and trapping. Led by the U.S. Geological Survey, the BTS RRT conducts annual trainings on and off Guam, maintains communication networks with agency partners and at-risk islands, conducts public outreach, and refines detection tools and deployment capabilities.

### Visual search detection estimate and assumption

Detection estimates were based on data from a censused population of marked snakes in a 5-ha snake enclosure on Andersen Air Force Base, Guam. The snake barrier prevents immigration and emigration of snakes from the forested site [11, 16]. The censused snake population has provided an opportunity to evaluate the effectiveness of control and detection tools including visual searching [10] and trapping [11], and to estimate the tool-specific detection probabilities for individual snakes under well-defined survey efforts along parallel transects at 8-m spacing. Over the past decade, research on Guam has shown that visual searching is an effective search tool for snakes of all sizes but detection probability does vary by size [10].

In this modeling exercise, we used a visual detection probability estimate (0.07) based on an average-sized snake (ca. 900 mm) of average body condition found under typical search conditions of no moonlight and low wind [10] and averaged over heterogeneous forest habitats. Visual detection estimates are directly related to search effort. In Guam, individual snake detection probability estimates on a given night were based on 5.94 km of searched transects (13.5 transects/night × 0.22 km/transect × 2 searchers/transect, each looking only to one side). We define this visual search unit from the Guam snake enclosure as 1 detection effort unit (DEU). Alternatively, if walking paces were similar to those used during detection estimation, one could specify 1 DEU in units of time (13.5 transects/night × 30 minutes/transect × 2 searchers/transect = 810 minutes or 13.5 hours). Because searchers tend to adjust their pace to the complexity of habitat (e.g., Guam 0.44 km/h vs. Rota 0.76 km/h) we felt that transect distance searched would be a better measure of effort for this system. Had Christy et al. [10] searched less intensively on a given night in the snake enclosure on Guam, fewer snakes would have been found and the individual-based detection probability estimate(s) would have been lower. Because we obtained almost identical estimates nearly 1.5 years later in this area when corrected for effort but after substantial forest regeneration [17] and higher snake densities, we feel that the detection estimate per visual search effort unit ($\hat{p}$ = 0.07) is robust.

We have limited data on snake detection as a function of its distance from a search transect, and that distance varies with vegetation structure. Applying visual detection rates derived from Guam to detection efforts on another island, therefore, relies on several assumptions. The fact that detection probabilities on Guam average 0.07 per night implies that snakes were rarely detected more than once per standardized visual search effort unit (i.e., 1 DEU), and detection of any particular individual was a binomial process rather than a count distribution process involving re-captures (i.e., should marked-and-released snakes have been captured more than once in a nightly 5.94-km survey effort). This allows extrapolation of detection probability from an intensive search of a dense population in a small area, to a less intense (i.e., geographically more spread-out) search effort in a modest-sized area where there may be an incipient snake population. This assumes that we confine searching to within the limits of an incipient population’s range and hypothesize the population density of snakes in that area.

We first assumed that the Rota visual search effort was conducted in an area where the snake population density was the same as in the snake enclosure (117 snakes in 5 ha, or ca. 24 snakes/ha) on Guam during the study of Christy et al. [10]. If so, then one person walking 5.94 km (1 DEU) and searching one side of a transect would likely find (0.07 × 117) = 8.19 snakes. We then decreased snake density (linear effect), increased search efforts (linear effect), and calculated a matrix of expected snake detections (Table 1).

**Table 1:** Expected numbers of detected brown treesnakes, given various visual search efforts and snake densities (individuals/ha), assuming that all snakes have the same detection probability as the average-sized snake had in the 5-ha snake enclosure on Guam (*p* = 0.07; [10]).

DEU	1	2	4	8	16	32	64	128	256
Kilometers searched	5.94	11.88	23.76	47.52	95.04	190.08	380.16	760.32	1520.64
Density									
24	8.19	16.38	32.76	65.52	131.04	262.08	524.16	1048.32	2096.64
16	5.46	10.92	21.84	43.68	87.36	174.72	349.44	698.88	1397.76
12	4.10	8.19	16.38	32.76	65.52	131.04	262.08	524.16	1048.32
8	2.73	5.46	10.92	21.84	43.68	87.36	174.72	349.44	698.88
6	2.05	4.10	8.19	16.38	32.76	65.52	131.04	262.08	524.16
4	1.37	2.73	5.46	10.92	21.84	43.68	87.36	174.72	349.44
3	1.02	2.05	4.10	8.19	16.38	32.76	65.52	131.04	262.08
2	0.68	1.37	2.73	5.46	10.92	21.84	43.68	87.36	174.72
1	0.34	0.68	1.37	2.73	5.46	10.92	21.84	43.68	87.36
0.5	0.17	0.34	0.68	1.37	2.73	5.46	10.92	21.84	43.68
0.25	0.09	0.17	0.34	0.68	1.37	2.73	5.46	10.92	21.84
0.1	0.03	0.07	0.14	0.27	0.55	1.09	2.18	4.37	8.74
0.05	0.02	0.03	0.07	0.14	0.27	0.55	1.09	2.18	4.37
(one snake/km^2^) 0.01	0.00	0.01	0.01	0.03	0.05	0.11	0.22	0.44	0.87

1 Detection Effort Unit (DEU) = 5.94 km of a one-sided transect search. The first expected value of 8.19 is based on *p* = 0.07 and 117 snakes known in the population (0.07 × 117).

### Poisson distribution application

Because the goal was to find evidence suggestive of an incipient population, this was the pressing question: What is the probability of finding at least one snake, given a specified search effort? To answer that question, we used a Poisson distribution and applied it to our estimates in Table 1. The Poisson distribution provides the probability of a given number of events arising in a fixed interval of time and/or space given that such events arise under the following two conditions: (1) a known average rate and (2) independence from one another [18]. Because the Poisson distribution is discrete, and based on integers (counts), we can, given a Poisson mean value (i.e., λ), calculate the probability of observing++ any snakes (i.e., finding 1, 2, 3, or more snakes). The Poisson distribution function calculates the probability of any observation *x* (*x* = 0, 1, 2…) as:

$$P\left(x\right)=e^{‑\lambda }\lambda^{x}/x!$$ (1)

where *e* is a constant (2.71828), the base of the natural logarithm. The probability of obtaining a count of zero snakes is obtained by setting *x* = 0:

$$P\;\left(0\;snakes\right)=e^{‑\lambda }.$$ (2)

If we consider an expected count of 8.19 snakes, λ = 8.19, inserted into Eq 2 we would obtain: *P*(0) = e^-8.19^ = 0.0002774. This estimate is the probability of finding 0 snakes when the expected snake count would be 8.19. Hence, the probability of finding >0 snakes is simply:

$$P\left(>0\;snakes\right)=1‑e^{‑\lambda }$$ (3)

which in our example is (1–0.0002774) = 0.9997226, or ca 99.97%. In Table 2, we apply Eq 3 to cell values from Table 1 to calculate the Poisson-based probabilities of finding >0 snakes.

**Table 2:** Poisson-based probabilities of detecting any snake (i.e., one or more snakes) given various visual detection efforts and snake densities (individuals/ha) based on calculations in Table 1.

DEU	1	2	4	8	16	32	64	128	256
Kilometers searched	5.94	11.88	23.76	47.52	95.04	190.08	380.16	760.32	1520.64
Density									
24	0.9997	1.0000	1.0000	1.0000	1.0000	1.0000	1.0000	1.0000	1.0000
16	0.9957	1.0000	1.0000	1.0000	1.0000	1.0000	1.0000	1.0000	1.0000
12	0.9833	0.9997	1.0000	1.0000	1.0000	1.0000	1.0000	1.0000	1.0000
8	0.9348	0.9957	1.0000	1.0000	1.0000	1.0000	1.0000	1.0000	1.0000
6	0.8709	0.9833	0.9997	1.0000	1.0000	1.0000	1.0000	1.0000	1.0000
4	0.7446	0.9348	0.9957	1.0000	1.0000	1.0000	1.0000	1.0000	1.0000
3	0.6408	0.8709	0.9833	0.9997	1.0000	1.0000	1.0000	1.0000	1.0000
2	0.4946	0.7446	0.9348	0.9957	1.0000	1.0000	1.0000	1.0000	1.0000
1	0.2891	0.4946	0.7446	0.9348	0.9957	1.0000	1.0000	1.0000	1.0000
0.5	0.1569	0.2891	0.4946	0.7446	0.9348	0.9957	1.0000	1.0000	1.0000
0.25	0.0818	0.1569	0.2891	0.4946	0.7446	0.9348	0.9957	1.0000	1.0000
0.1	0.0335	0.0660	0.1276	0.2389	0.4207	0.6645	0.8874	0.9873	0.9998
0.05	0.0169	0.0335	0.0660	0.1276	0.2389	0.4207	0.6645	0.8874	0.9873
(one snake/km^2^) 0.01	0.0034	0.0068	0.0136	0.0269	0.0531	0.1034	0.1962	0.3539	0.5826

To generate these values we applied Eq 3 to each cell value in Table 1. The first expected value was calculated as *P*(> 0) = 1—e^-8.19^ = 1–0.0002774 = 0.9997226. This matrix clearly illustrates that the amount of effort necessary to declare that a species is absent is substantial and increases with decreased snake density and increasing level of confidence about its absence. The total survey effort required also increases if the individual detection probability (for a survey effort of 1 DEU) is lower than the *p* = 0.07 here assumed for illustrative purposes.

The conditions on Guam (dense populations of snakes) would be more favorable for detection by visual searchers than would snakes in an incipient population on another island, where they might be well fed from the greater abundance of prey [19]. While we lack information to precisely predict how detection would manifest itself on a new island where snakes might get established, we can safely assume that conditions in a new population will be less favorable for detection than those documented on Guam ([10]; *p* = 0.07). We therefore assumed a 50 percent reduction in snake detection because well-fed snakes are more frequently satiated and inactive [20] and then produced predictions for Rota by simply multiplying the expected snake counts in Table 1 with a specified detection ratio *p*_Rota_/ *p*_Guam_ (e.g., 0.035/0.07 = 0.5). Thus, the first expected value at 24 snakes/ha for 1 DEU would now yield 4.095 snakes (8.19 × 0.5) in Table 1. When this adjustment is applied to a corresponding table of probabilities of detecting any snake, our example of of 4.095 snakes would become *p*_Rota_ = 0.9833, or 98.33% chance of detecting any snake (as opposed to the 99.97% chance at a detection probability of *p*_Guam_ = 0.07 actually listed in Table 2). Below, we use the actual EDRR visual search effort (639.4 km) implemented by the BTS RRT in 2014 to demonstrate Poisson model application. Another capture method, mouse-baited snake traps, is demonstrated (S1 Table, tab = EDRR trap effort) and discussed (S1 Text) in Supplementary materials. We present Poisson-based probabilities of detecting any snake over a range of plausible individual detection probabilities (0.01–0.07; from worst- to best-case scenarios) and snake densities (24 to 0.01/ha) in order to capture the inherent uncertainty of these estimates (Tables 3 and 4).

**Table 3:** Expected numbers of snakes to be found in 639.4 km of one-sided visual surveys at night, given various detection probabilities (*p*) and snake densities (individuals/ha).

Individual snake detection probability	0.07	0.06	0.05	0.04	0.035	0.03	0.02	0.01
Density								
24	881.60	755.65	629.71	503.77	**440.80**	377.83	251.88	125.94
16	587.73	503.77	419.81	335.85	**293.87**	251.88	167.92	83.96
12	440.80	377.83	314.86	251.88	**220.40**	188.91	125.94	62.97
8	293.87	251.88	209.90	167.92	**146.93**	125.94	83.96	41.98
6	220.40	188.91	157.43	125.94	**110.20**	94.46	62.97	31.49
4	146.93	125.94	104.95	83.96	**73.47**	62.97	41.98	20.99
3	110.20	94.46	78.71	62.97	**55.10**	47.23	31.49	15.74
2	73.47	62.97	52.48	41.98	**36.73**	31.49	20.99	10.50
1	36.73	31.49	26.24	20.99	**18.37**	15.74	10.50	5.25
0.5	18.37	15.74	13.12	10.50	**9.18**	7.87	5.25	2.62
0.25	9.18	7.87	6.56	5.25	**4.59**	3.94	2.62	1.31
0.1	3.67	3.15	2.62	2.10	**1.84**	1.57	1.05	0.52
0.05	1.84	1.57	1.31	1.05	**0.92**	0.79	0.52	0.26
(one snake/ km^2^) 0.01	0.37	0.31	0.26	0.21	**0.18**	0.16	0.10	0.05

A plausible estimate for *p* on Rota (0.035) is shown in bold font and is half of what we would expect for snakes seen on Guam (*p* = 0.07). This particular effort (640 km) reflects the actual kilometers searched during the 2014 Rota EDRR deployment. Cell values in this table would change depending on the number of kilometers.

**Table 4:** Poisson-based probabilities of detecting any snake (i.e., one or more snakes) in 639.4 km of one-sided visual searches at night, given various detection probabilities and snake densities (individuals/ha).

Individual snake detection probability	0.07	0.06	0.05	0.04	0.035	0.03	0.02	0.01
Density								
24	1.0000	1.0000	1.0000	1.0000	**1.0000**	1.0000	1.0000	1.0000
16	1.0000	1.0000	1.0000	1.0000	**1.0000**	1.0000	1.0000	1.0000
12	1.0000	1.0000	1.0000	1.0000	**1.0000**	1.0000	1.0000	1.0000
8	1.0000	1.0000	1.0000	1.0000	**1.0000**	1.0000	1.0000	1.0000
6	1.0000	1.0000	1.0000	1.0000	**1.0000**	1.0000	1.0000	1.0000
4	1.0000	1.0000	1.0000	1.0000	**1.0000**	1.0000	1.0000	1.0000
3	1.0000	1.0000	1.0000	1.0000	**1.0000**	1.0000	1.0000	1.0000
2	1.0000	1.0000	1.0000	1.0000	**1.0000**	1.0000	1.0000	1.0000
1	1.0000	1.0000	1.0000	1.0000	**1.0000**	1.0000	1.0000	0.9947
0.5	1.0000	1.0000	1.0000	1.0000	**0.9999**	0.9996	0.9947	0.9275
0.25	0.9999	0.9996	0.9986	0.9947	**0.9899**	0.9805	0.9275	0.7307
0.1	0.9746	0.9571	0.9275	0.8774	**0.8407**	0.7928	0.6499	0.4083
0.05	0.8407	0.7928	0.7307	0.6499	**0.6008**	0.5449	0.4083	0.2308
(one snake/ km^2^) 0.01	0.3074	0.2701	0.2308	0.1893	**0.1678**	0.1457	0.0996	0.0511

A plausible estimate for *p* on Rota (0.035) is shown in bold font. Probabilities were generated from applying Eq 3 to each corresponding value from Table 3. For example, the expected value (assuming a visual detection of 0.035) at a snake density of 0.25/ha was calculated as *P*(> 0) = 1—e^-4.59^ = 1–0.010153 = 0.9899.

## Results

To capture uncertainty and facilitate decision making for the level of effort needed to infer an absence, we modeled the probabilities of detecting any snake over a range of likely visual detection estimates, snake densities, and specified DEUs (km). Matrices provided in Supplementary material (S1 Table) illustrate the substantial amount of effort required to declare that a species is absent and how the response effort increases with decreased individual detection probability, decreased population density, and increased level of desired confidence about its absence. These tradeoffs are readily observed in Table 2: at a snake density of 0.05/ha (i.e., 5 snakes/km^2^), there is a 1.7% chance of finding any snake during one DEU (5.94 km), but as distance increases to 256 DEU (1,520 km) the probability increases to 98.7%. To easily evaluate various levels of effort and associated confidence, we programed a data cell for number of kilometers searched so that as the value is manipulated, new Poisson probabilities populate cells in corresponding matrices provided in Supplementary material (S1 Table, tab = EDRR Visual search effort).

Expected numbers of snakes to be found in 639.4 km of one-sided visual surveys at night (actual 2014 effort), given various detection probabilities (*p*; 0.01–0.07) and snake densities (0.01-24/ha) ranged widely from 0.05 to 881.60 (Table 3). A plausible detection probability for snakes, if they were to occur, on Rota is ca. 0.035 (Table 3, boldface) and is associated with expected snake numbers ranging between 0.18–444.80, at densities of 0.01 to 24 snakes/ha, respectively. When Eq 3 is applied to values in Table 3, we obtain the Poisson-based probabilities of detecting any snake (i.e., one or more snakes) in 639.4 km of one-sided visual searches at night, given various detection probabilities and snake densities (Table 4). Given this substantial effort, we would have detected at least one snake (with a confidence level exceeding 99%) in the search area had the density been 1 snake/ha, irrespective of detection probabilities (0.01–0.07). Here we also see that if a brown treesnake population in the searched area had been about one-hundredth as dense as that in Guam (0.25 snakes/ha compared to ca. 20–25 snakes/ha), the rapid response effort (639.4 km) would have visually detected one or more snakes with a probability of 99% (Table 4; column *p* = 0.035). For a 95% probability, we can easily interpolate the probability estimate between the snake densities of 0.1 (0.8407) and 0.25 (0.9899) snakes in Table 4. This interpolation would allow a manager to make the following statement: Based on the visual search effort of 639.4 km, there is a 95% probability that the rapid response effort in this area would have visually detected at least one snake, had the population density been 0.16 snakes/ha or higher and assuming an individual detection probability of 0.035 per DEU.

## Discussion

Successful early detection and rapid response efforts for incipient invasive populations require infrastructure, proven detection/control tools, readiness to respond, immediate access to adequate funding [21] and analytical and decision tools [7], ideally combined with digital data collection [22]. Of these components, analytical and decision support tools have historically received the least attention. Indeed, prior to our modeling effort, there had been no quantification of brown treesnake rapid response efficacy or duration despite numerous sightings in the Pacific region [15] of this highly invasive species. Over the past decade there has been an increase in applied analytical and decision support tools for EDRR across taxa that include optimizing surveillance programs [23–28] and related questions of deciding when to declare a successful eradication of an established invasive species [25, 29–34], generating model-based forecasts of invasions [35, 36], and sampling to detect rare species [37, 38].

In our modeling framework we assessed the probability that additional snakes will be found during surveys, should there be a snake population present in the focal area. We developed this model based on effort-linked detection probabilities from a censused brown treesnake population on Guam and calculated the probability that the finite rapid response effort would have revealed any snake. We adjusted detection probabilities from Guam downward to account for Rota’s more prey-rich environment and then estimated the probability of encountering any snake(s) under a wide range of hypothetical population densities. Unlike past rapid response efforts for this species, we can now conduct an analysis grounded in rigorous ecological research in order to provide resource managers with the desired level of confidence for a given deployment effort. We found that had the brown treesnake population density in the surveyed area been ≥0.25/ha the deployment should have detected one or more snakes with a probability exceeding 99% given the search effort of 639.4 km and an individual snake detection probability of 0.035 per DEU. Our matrices readily calculate probabilities for inferring an absence given varying levels of search effort over a range of individual snake detection probabilities (see S1 Table). While we have estimated the probability of inferring the absence of an incipient population, we acknowledge that it is the decision of regulatory agencies and associated natural resource managers to determine the level of certainty and what is the reasonable level of doubt. This decision is a tradeoff between (1) declaring that no incipient population exists with the risk that the species is present and can continue to grow and spread and (2) continuing to survey with the risk that the species is not established and limited resources are wasted. Poisson detection probability matrices clearly show, as one would expect, the slim probability of detecting average-sized snakes when snake density is low and that the effort to declare the absence of a species requires a substantial investment of time and resources. Based on our findings, the minimum sampling effort for inferring an absence may be greater than previously estimated for cryptic species [39, 40] or rare species [37] and clearly underscores the need to increase detectability of such species when feasible.

As with all models there are assumptions. In our framework we used detection estimates for our case study species from a known population on Guam in which the detection probability of a snake of average size, sex, and body condition under modal environmental conditions and across varied microhabitats was *p* = 0.07, but snake size and other variables are known to cause individual variation [10]. Do we need to be concerned about such individual variation when predicting our likelihood of finding evidence for an incipient population? Yes and no. Ultimately, we think “no” because in our framework, we are not looking at how easy it would be to *remove* the *last* snake (which should be the hardest one to detect) of a hypothetical snake population. We are merely asking if our search effort is sufficient to detect *any* snake. With variation around average detection probability, some snakes would be easier to find, some would be harder to find, and many will be found with the average probability. The question then becomes this: Is the detection probability effect linear, so that the easier-to-find individuals make up perfectly for the harder-to-find individuals, causing the average detection that we use in our modeling to render an unbiased answer? For this question, we think so. But the answer to our original question might also be “yes” if the detectability varies among individuals with particular traits, and if the distribution of those traits are different in an incipient population compared to the population where the detection probability was estimated. We adjusted for an assumed systematic population bias in snake satiation by simply multiplying the assumed detection probability in Rota by 0.5. Similar adjustments might be desirable if traits such as sex, size, body condition, etc., affect detection probability, and if those traits are assumed to differ between established and incipient populations due to factors such as habitat variability. Hence the variation itself is not an issue, but a difference in the population means might be problematic. Adjusting detection probabilities for hypothetical population traits is likely to present a challenge but we err on the side of conservatism and present confidence estimates over a range of detection probabilities.

In our case study we lacked specific estimates for searching in low snake density situations and had to make assumptions. The detection estimates from Christy et al. [10] and Tyrrell et al. [11] come from dense snake populations. A rarity of targets would likely diminish searcher efficacy due to a lower positive reinforcement rate [41], although experienced searchers such as those participating in BTS RRT deployments are presumably more likely to maintain search effectiveness in the absence of frequent rewards. MacKenzie et al. [42] advocate that information on detectability be ‘borrowed’ or inferred from other times, locations, or even species; therefore, we think it is reasonable to extend our model to other species when surrogate density-specific estimates are available. When detection probabilities cannot be reasonably borrowed from the literature, managers could use other options: (1) expert opinion could be solicited to define a range of plausible detection estimates [43–45], or (2) crude detection information on a focal taxon could be collected in ways less labor intensive than were obtained for our focal species by Christy et al. [10]. For the latter option, proxies for the focal taxon could be placed at random but known locations for searchers to find [46, 47] or an area occupied by telemetered individuals could be surveyed by “blind” searchers [48]. Such work could be conducted in areas where the focal taxon is already established, rather than in the area where an incipient population is suspected. We recognize that species-specific estimates are most critical for species that are highly invasive but hard to detect, and therefore have a high potential to spread to other sensitive areas; snakes therefore present a very suitable test case [5, 49] for development of our analytical approach.

Our analysis relies on the assumption that we are searching in the correct area, i.e., where an incipient population would be expected (here, searching the vicinity around a credible sighting). Inferences are limited to the search area, although brown treesnakes are habitat generalists and virtually all areas on recipient islands consist of suitable snake habitat. As long as the organism is likely to be distributed across the search area, and somewhat mobile, the size of the area or how transects are laid out does not matter—only the effort matters. We are equally likely to run into *any* snake if we concentrate our searching to a 1-ha area, as if we were to search one-tenth as intensely over a 10-ha area. A larger area means a lower individual detection probability, but this is offset by the higher number of targets in a larger area.

For intensive surveys in a small area, the probability of detecting one *particular* snake that currently happens to be present is high, but there are likely to be few available targets. Indeed, the snake density may be so low that there is probably no snake at all in the survey area on any given night, although random snake movements across the landscape would eventually cause them to pass through the survey area. The cumulative probability of detecting *any* snake is therefore the same, should we spread our effort over a larger area. This obviously applies to organisms that are somewhat mobile, and where repeated surveying of the same transects over time is not a duplicative and wasted effort.

Whereas our model is meant to assess if an incipient population is established in a larger area, a rapid response elicited by a recent sighting at a well-defined location might argue for centering the search area within that locale. All searched transects in the 2014 Rota EDRR effort were within a 47-ha area surrounding the Rota seaport (where a snake had been captured). We surveyed 639.4 km, rendering a 95% chance of detecting a snake population of 0.16 individuals/ha. Hence our 95% probability corresponds to no more than (0.16 snakes/ha × 47 ha) = 7.5 snakes being present in the search area on an average night. Given a fixed search effort and a specific confidence level, the larger the search area (over which the effort is diluted) the more snake individuals could have gone undetected. That is simply because for a particular organism density, *N* scales linearly to area. If responding to multiple sightings of an invasive species over a larger spatial extent, then a different sampling strategy (e.g., stratified random sampling [50] or adaptive sampling [51]) would be warranted. Our model should apply to different sampling approaches.

Cost-benefit [23] and population demography extensions to our modeling framework could be incorporated (if known) and would further increase the utility of our analytical tool. For instance, if cost is estimated for one unit of detection effort (DEU), then managers working within a specified budget could predict costs associated with various levels of certainty. Using our case study with a hypothetical monetary estimate of US$100/DEU, we could then state the following: snake density would need to be at or lower than 0.16 snakes/ha or else the visual searches (639.4 km) would have turned up at least one snake with 95% probability and such an effort would cost US$10,746 ((639.4 km/(5.94 km/DEU)) × (US$100/DEU)).

Finally, if one has prior knowledge of the intrinsic rate of growth in an incipient population of the focal species, and one were to make assumptions of the current population density and demography, one could envision modeling the growth of a suspected, but yet unverified, population. This might allow estimation of the (higher) probability of detecting any population during a repeated survey at some time in the future—which would come at a cheaper labor effort, but occur when the potentially invasive organism has gained an even stronger foothold in its new range.

## Supporting information

S1 Table Poisson probability matrices for visual search (described in this paper) and trapping effort (narrative and assumptions provided in S1 Text) are programmed into a spreadsheet. (XLSX)

S1 Text Trap Capture Narrative: Estimation and Assumptions. (DOCX)
