# Supplementary material for: Inferring the absence of an incipient population during a rapid response for an invasive species
Source: PLoS One. 2018 Sep 27;13(9):e0204302. doi: 10.1371/journal.pone.0204302 (PMC6160030; doi:10.1371/journal.pone.0204302)
Supplement: S1 Text — (DOCX) [file pone.0204302.s002.docx]

## S1 Text. Trap Capture Estimation and Assumptions

Here, we model the probability of finding ≥1 snake(s) based on two parameters: the potential snake population density in the surveyed area, and detection probability estimates (for individual snakes, given a particular survey effort) associated with snake trapping. Brown treesnake (*Boiga irregularis*) traps are constructed of galvanized steel mesh with a sloping metal mesh flap, covered with plastic shade protection and fitted with a live lure mouse protected inside a mesh chamber. Mouse-baited traps for brown treesnakes are highly effective for adult snakes but minimally so for small snakes; therefore modeling described in this Supplementary material (S1 Table, S1 Text) addresses the population segment that would be large enough to readily enter a trap. The switch from low to high trappability occurs over the 700 – 900 mm snout-vent length interval [1, 2]. Most control tools on Guam are conducted in a prey-limited system; this is not the case on Rota [3], so we adjusted expected trap capture [4] downward in our detection scenarios and generated matrices of the probabilities that specified trap efforts will find at least one snake over a range of population density by detectability combinations.

In the 5-ha snake enclosure on Guam, an array of 13 × 13 = 169 traps are used [2]. Tyrrell et al. [2] estimated the nightly mean detection probability of a snake by trapping to be *p* = 0.14. That estimate, however, is only valid for an average-sized (size interval 900 – 999 mm SVL) female of average body condition (in the enclosure at the time), under the most frequently experienced wind, rain, and moon conditions. Snakes below an ill-defined size of 700 – 900 mm snout-vent length have extremely low trap capture probabilities [2]. Applying these data to a hypothetical snake population offers several challenges, most notably the following: 1) The demography (size distribution) of an incipient population is unknown; 2) the body condition indices of hypothetical incipient snakes are unknown; and 3) the trapping intensity (traps per unit area) is not always the same on deployments as in the snake enclosure. Related to item (2) listed above is a short-term satiation effect that we expect snakes in prey-rich locales to experience more frequently than do snakes in prey-depleted Guam. Satiation is expected to make snakes less inclined to forage and enter traps.

Denser trap spacing (as in the enclosure 16 m × 16 m) means that traps “compete” with each other for the same snakes more than traps deployed during a typical brown treesnake rapid response (20 m to 40 m spacing), therefore we would anticipate that detection probability is higher with the somewhat less dense deployment trap spacing. But this effect is probably outweighed by the high prey density, and in particular high rodent densities, found on neighboring islands in this region. Rat densities on Rota have ranged from 36 to 96 rats / hectare compared to densities of 0.7 to 16 rats / hectare for similar habitats on Guam [3]. Gragg et al. [4] demonstrated that when rats were eliminated from four grassland plots in southern Guam, snake trappability increased by 22 to 65% the following week. Rats were somewhat scarce at the time of the Tyrrell et al. [2] trapping study, and the rat densities on adjacent islands are presumably even more different from the snake enclosure than are the rat density differences between the enclosure and grassland sites of southern Guam [cf. 3]. Therefore, we reason that our capture probability of 0.14 from a prey-limited trap area should be adjusted downward by 65%; (1 - 0.65) = 0.35, and thus *p* = (0.14 × 0.35) ≈ 0.05. This hypothetical rapid response deployment trap-capture probability of 0.05 is quite similar to the average trap-capture probability (0.07) prior to rodenticide treatment application in south Guam [4].

As with visual search data, we captured uncertainty and facilitated decision making by creating probability matrices of detecting any snake over a range of capture estimates, snake densities, and specified effort unit (trap nights). Matrices found in Supplementary materials (S1 Table, see EDRR Trap effort) illustrate the substantial amount of effort required to declare that a species is absent and how the response effort increases with decreased trap capture, decreased density, and a highly desired level of confidence about its absence. To evaluate various levels of effort and associated confidence, we programed a data cell for number of trap nights so that as the value is manipulated new Poisson probabilities populate in corresponding matrices in Supplementary materials (S1 Table, see EDDR Trap effort).

**References**

1. Rodda GH, Savidge JA, Tyrrell CL, Christy MT, Ellingson AR. Size bias in visual searches and trapping of brown treesnakes on Guam. Journal of Wildlife Management. 2007;71(2):656-61.

2. Tyrrell CL, Christy MT, Rodda GH, Yackel Adams AA, Ellingson AR, Savidge JA, et al. Evaluation of trap capture in a geographically closed population of brown treesnakes on Guam. Journal of Applied Ecology. 2009;46(1):128-35.

3. Wiewel AS, Yackel Adams AA, Rodda GH. Distribution, density, and biomass of introduced small mammals in the southern Mariana Islands. Pacific Science. 2009;63(2):205-22.

4. Gragg JE, Rodda GH, Savidge JA, White GC, Dean-Bradley K, Ellingson AR. Response of brown treesnakes to reduction of their rodent prey. Journal of Wildlife Management. 2007;71(7):2311-7.
